# Mechanochemistry of nucleosides, nucleotides and related materials

**DOI:** 10.3762/bjoc.14.81

**Published:** 2018-04-27

**Authors:** Olga Eguaogie, Joseph S Vyle, Patrick F Conlon, Manuela A Gîlea, Yipei Liang

**Affiliations:** 1 School of Chemistry and Chemical Engineering, Queen’s University Belfast, David Keir Building, Stranmillis Road, Belfast BT9 5AG, UK

**Keywords:** DNA, green chemistry, mechanochemistry, nucleoside, nucleotide

## Abstract

The application of mechanical force to induce the formation and cleavage of covalent bonds is a rapidly developing field within organic chemistry which has particular value in reducing or eliminating solvent usage, enhancing reaction rates and also in enabling the preparation of products which are otherwise inaccessible under solution-phase conditions. Mechanochemistry has also found recent attention in materials chemistry and API formulation during which rearrangement of non-covalent interactions give rise to functional products. However, this has been known to nucleic acids science almost since its inception in the late nineteenth century when Miescher exploited grinding to facilitate disaggregation of DNA from tightly bound proteins through selective denaturation of the latter. Despite the wide application of ball milling to amino acid chemistry, there have been limited reports of mechanochemical transformations involving nucleoside or nucleotide substrates on preparative scales. A survey of these reactions is provided, the majority of which have used a mixer ball mill and display an almost universal requirement for liquid to be present within the grinding vessel. Mechanochemistry of charged nucleotide substrates, in particular, provides considerable benefits both in terms of efficiency (reducing total processing times from weeks to hours) and by minimising exposure to aqueous conditions, access to previously elusive materials. In the absence of large quantities of solvent and heating, side-reactions can be reduced or eliminated. The central contribution of mechanochemistry (and specifically, ball milling) to the isolation of biologically active materials derived from nuclei by grinding will also be outlined. Finally non-covalent associative processes involving nucleic acids and related materials using mechanochemistry will be described: specifically, solid solutions, cocrystals, polymorph transitions, carbon nanotube dissolution and inclusion complex formation.

## Introduction

Several definitions of mechanochemistry have been attempted since Ostwald included it as one of four taxa along with thermochemistry, electrochemistry and photochemistry [1]. A general definition commonly cited is that developed by The International Union of Pure and Applied Chemistry (IUPAC) to encompass both the chemical and physical effects of shearing, stretching or grinding polymeric materials: "[a mechano-chemical reaction is one] induced by the direct absorption of mechanical energy" [2]. The etymology and early history of this field have been reviewed comprehensively by Takacs [1]. Several recent reviews discuss both general aspects of mechanochemistry [3–4] as well as more focussed elements of the subject relevant to the current work including applications in organic synthesis [5–8], green chemistry [9], API formulation [10] and coordination/materials chemistry [11–12]. Some aspects of the current work have also been reviewed recently [13]. However, the impact of mechanochemistry upon biological chemistry and specifically the selective degradation of biopolymers which enables biochemically active materials to be isolated from cell grindates – most notably in Buchner’s laboratory [14] – appears not to have been considered.

A recent tutorial review by Andersen and Mack [15] augments an earlier introduction describing both the parameters used to define such chemistry and also how this information is conveyed in synthetic schemes [16]. Stolle has written a comprehensive treatise on the chemical, technological and process parameters which influence the outcome of a ball mill reaction [17]. In this review we have also adopted Hanusa’s formalism which distinguishes ball milling from other forms of mechanochemistry [18].

**Figure 1:**
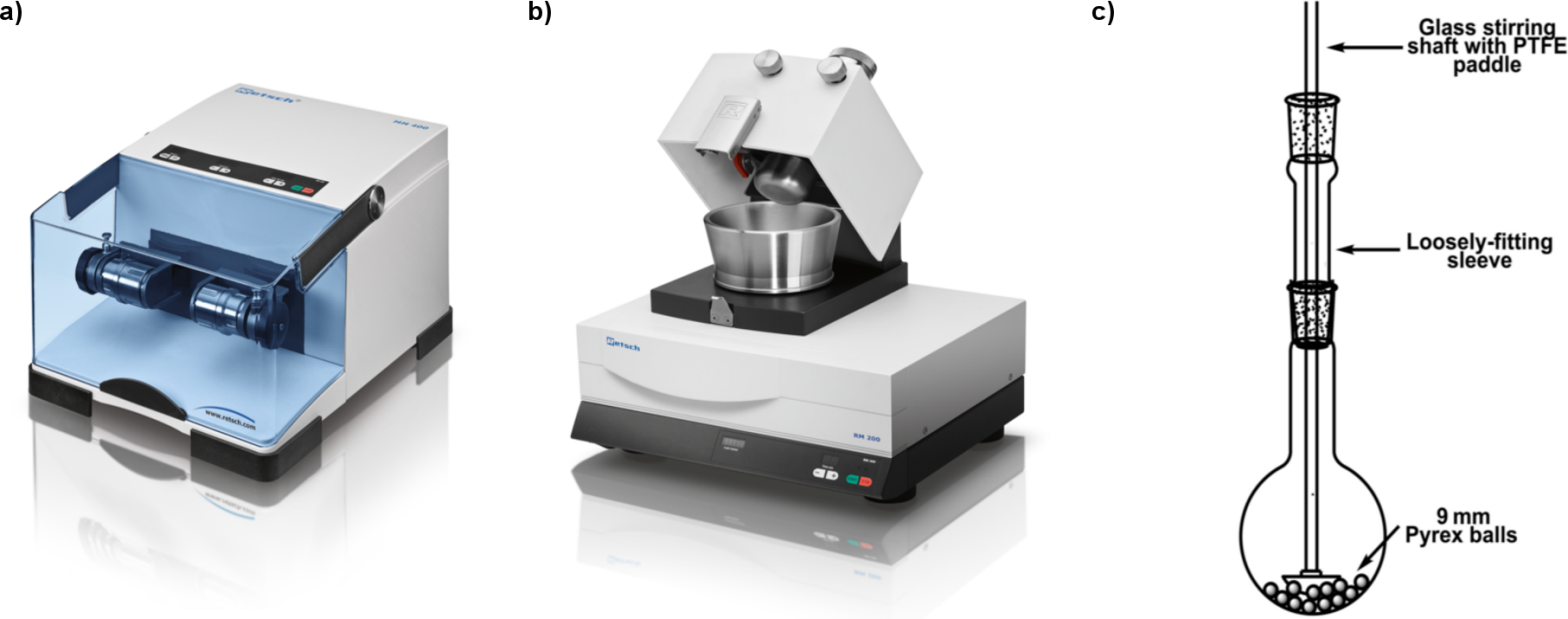
Examples of equipment used to perform mechanochemistry on nucleoside and nucleotide substrates (not to scale). a) Mixer ball mill; b) mortar grinder; c) improvised attritor [19]. Figures a) and b) are reused with the permission of Retsch (https://www.retsch.com); c) is adapted with permission from [19], copyright 2006 American Chemical Society.

Perhaps most critical to the recent interest in this field has been the ability to deliver consistent and reproducible levels of mechanical energy using commercially-available equipment which, for reactions of nucleosides and related materials has most commonly been the mixer ball mill (MBM – e.g., Figure 1a). Using a MBM, high energy collisions between reactants and one or more balls within a closed vessel (jar) are induced by vibrating the jar through a limited arc (ca. 0.5°) within one plane at up to 60 Hz (more typically 30 Hz). In its single-armed form, this is sometimes referred to as an amalgum mill. Alternatively, grinding actions have been provided using a mechanised mortar mill which mimics the action of hand grinding in a mortar and pestle (Figure 1b), an improvised attritor-type device (Figure 1c) or a planetary ball mill (not shown).

The amount of mechanical energy delivered to the reaction mixture via these collisions is a function of several engineering parameters including: the frequency of vibration; the degree of filling of the vessel (and its shape); the mass of the ball(s); and the hardness of the colliding materials. In order of descending hardness, zirconia, stainless steel, copper and PTFE have all been used to effect mechanochemical transformation of nucleoside or nucleotide substrates. During a study of amide coupling under ball-milling conditions, Lamaty and co-workers showed that deterioration of vessels and balls by physical abrasion and/or chemical leaching gave products in which (depending upon the nature of the jar) iron, chromium, zirconia or PTFE were detected [20]. This has influenced the choice of vessel for nucleoside and nucleotide chemistry as, although considerably cheaper, leaching of iron from stainless steel vessels in the presence of sulfur-containing materials [21] has been found to inhibit the preparation of thionucleoside [22] or thionucleotide [23] analogues. Although grinding using PTFE components delivers less energy due to the material’s elasticity and low density (2.1 g cm^−3^) compared with stainless steel (7.8 g cm^−3^) or zirconia (5.9 g cm^−3^), PTFE may be required for the preparation of pharmaceutical grade materials which are subject to regulatory approval.

Theoretical models of mechanochemical bond activation are mainly based upon examination and/or modelling the behaviour of single molecules under tension in an atomic force microscope [24–26] and have included relating traditional Arrhenius reaction parameters to applied forces [27–28]. However, early models of macroscopic scale reactivity in the solid-state (such as the formation of a short-lived plasma phase [29]) do not account for observations on the comminution of organic reactants during milling such as changes to the physical form of the mixture (including zones of liquefaction [30] and cohesive states [31]) which are correlated with the progress of the reaction including induction periods of up to 40 minutes [31–32]. Rate enhancements may thus be achieved from very high localised reactant concentrations within which developing intermolecular and intramolecular interactions are formed that can lead to reaction of a nucleoside or nucleotide substrate which would be disfavoured in solution. Although bond disruption via ultrasound-induced cavitation can be considered within the purview of mechanochemistry [33–34], this review will be restricted to the delivery of mechanochemical energy on a macroscopic scale by vibration, grinding and/or crushing actions. Furthermore, the term grinding will be applied throughout even though kneading (often referred to as solvent-drop grinding) is more accurate to describe the process of grinding or milling mixtures of solids and liquids [35]. To date, all but one chemical transformation of solid nucleoside or nucleotide substrates have been performed in the presence of liquids. These may originate either from the use of reagents which are liquids or low-melting solids (which liquify upon grinding) or from the addition of stoichiometric quantities of molecular solvents (or ionic liquids). During subsequent discussions, liquid-assisted grinding (LAG) is used to describe only the latter case.

**Figure 2:**
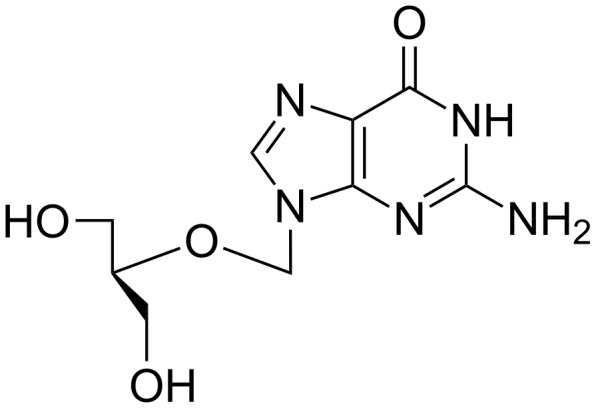
Ganciclovir.

The minimal level of solvent requirement is particularly advantageous in the context of charged nucleotide substrates as considerable cost, time and energy savings can be gained in the absence of arduous ion-exchange and drying processes required to render these materials soluble in organic solvents. Likewise, significant reductions in solvent processing (especially if these are high boiling and often toxic and/or carcinogenic) is an attractive green chemistry target. In this context, Thorwith et al. compared the amount of energy required to effect permanganate-mediated oxidative self-coupling of *p*-toluidine using different energy inputs. Ball milling was significantly more efficient (up to an order of magnitude) than conventional heating, microwave or ultrasound inputs [36]. The reduction in both solvent and energy input are particularly relevant in fine chemical manufacturing processes which typically have very high E-factors and low energy efficiency [37]. Although mechanochemistry was not involved in redesigning the synthesis of the antiviral prodrug ganciclovir (Figure 2), the high levels of involatile solvent usage typically employed in the solution-based synthesis of such compounds can be gauged by the ability of Roche to eliminate 1.12 million kilograms of solvent per annum [38].

## Review

### Mechanochemical transformations of nucleosides and related materials involving covalent bonds

#### Reactions of nucleoside sugar and nucleobase moieties

**Scheme 1:**
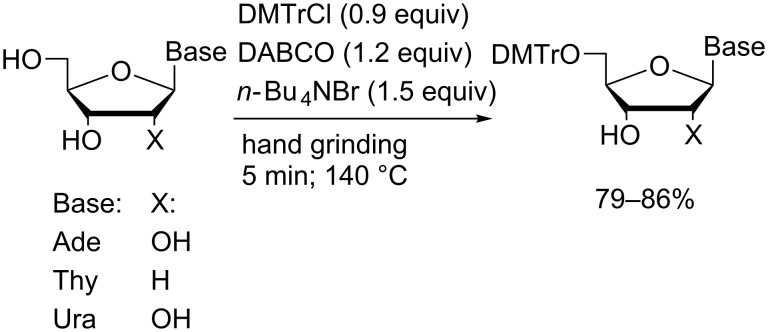
Nucleoside tritylation effected by hand grinding in a heated mortar and pestle.

An early example of the application of mechanochemistry for nucleoside derivatisation was reported by Khalafi-Nezhad and Mokhtari who effected regioselective 5′-protection of ribonucleosides and thymidine using a mortar and pestle with trityl-, monomethoxytrityl- or dimethoxytrityl chloride (Scheme 1) [39].

A variety of temperatures and either inorganic or low-melting organic bases were surveyed. Optimal yields were achieved at 140 °C using DABCO by hand-grinding the reaction mixture in molten tetra-*n*-butylammonium bromide (TBAB) for five minutes. In the presence of excess nucleoside (1.1 equiv), the corresponding 5′-trityl ethers of uridine, adenosine or thymidine were isolated in yields up to 86%. Reactions of guanosine or cytidine under these conditions gave rise to mixtures of products from which the corresponding tritylated products could not be isolated.

Subsequently, Patil and Kartha described the gram-scale preparation of 5′-tritylated uridine derivatives in a planetary ball mill (using a steel vessel and balls) in the absence of TBAB [40]. Following extended grinding (600 rpm for 15 hours) of the nucleoside in the presence of excess DABCO and either TrCl or DMTrCl, the products were recovered in 44% and 43% yields, respectively.

**Scheme 2:**
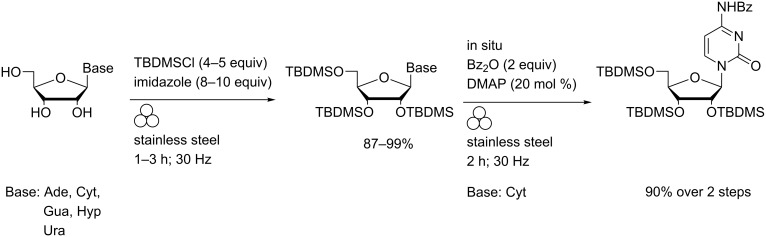
Persilylation of ribonucleoside hydroxy groups (and in situ acylation of cytidine) in a MBM.

Under solvent-free Corey conditions, rapid and chemoselective persilylation of ribonucleoside hydroxy functions was effected in a mixer ball mill (Scheme 2) [41].

Complete consumption of starting materials was observed within one to three hours and only in the case of adenosine was any (minor) side-product formation found. In all cases, facile purification using a scrubber column enabled pure 2′,3′,5′-tri-*O*-TBDMS-protected nucleosides to be isolated in 87–99% yields. In situ benzoylation of the persilylated cytosine was also effected following addition of benzoic anhydride and catalytic DMAP to the crude reaction mixture and extending the milling time. Quantitative silylation of 5′-*O*-dimethoxytritylthymidine under these conditions was also reported.

**Scheme 3:**
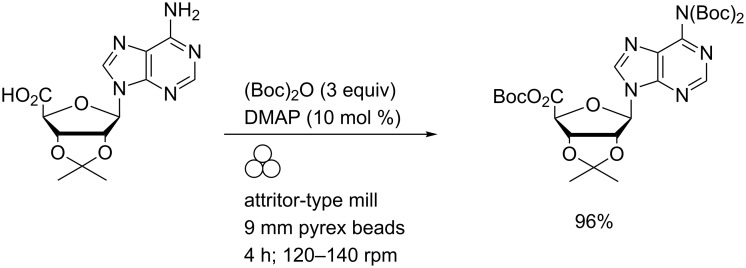
Nucleoside amine and carboxylic acid Boc protection using an improvised attritor-type mill.

Prompted by the insolubility of adenosine 5′-carboxylic acid derivatives, Sikchi and Hultin contrived an attritor-type mill (Figure 1c) to facilitate the use of neat reagents and vent CO_2_ (closed vessels were reported to break) [19]. Efficient gram-scale Boc protection of amine and carboxylic acid functions was thereby effected (Scheme 3).

This chemistry was further applied to the derivatisation of the exocyclic amino functions of hydroxy-protected adenosine and cytidine derivatives (and the corresponding 2′-deoxynucleosides). The majority of these reactions proceeded in excellent yields (90–99%) over one to six hours. In contrast, guanine-derived (deoxy)nucleosides generally required longer to achieve complete reaction and yielded the corresponding *O*^6^,*N*^2^,*N*^2^-tri-Boc derivatives with variable recoveries (25–70%).

**Scheme 4:**
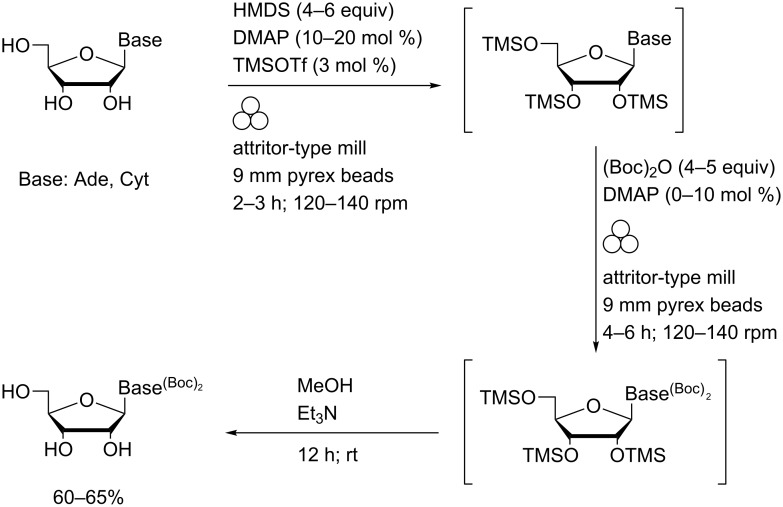
Nucleobase Boc protection via transient silylation using an improvised attritor-type mill.

The scope of this reaction was extended to unprotected nucleosides by effecting a one-pot, two-step reaction sequence (Scheme 4). Initial transient silylation and subsequent Boc-protection were both performed in the absence of solvent under mechanochemical conditions. In situ methanolysis of the TMS ethers yielded the corresponding base-protected nucleosides.

**Scheme 5:**
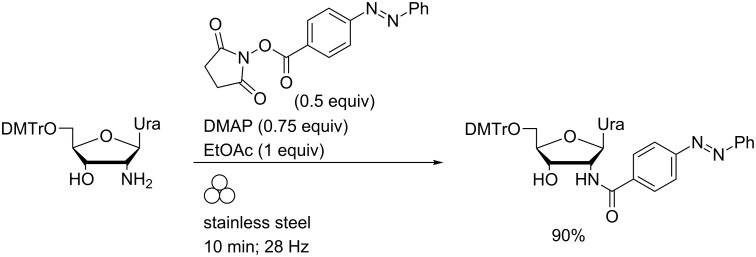
Chemoselective *N*-acylation of an aminonucleoside using LAG in a MBM.

The opaque nature of typical reaction vessels used in ball milling has enabled cleaner reactions of nucleoside analogues with photoreactive materials. Thus, liquid-assisted grinding of the *N*-hydroxysuccinimidyl esters of *o*-, *m*- or *p*-phenylazobenzoic acids with excess D-threoninol or of the *para* isomer with an aminonucleoside in the presence of DMAP and ethyl acetate engendered chemoselective *N*-acylation (Scheme 5) [42]. In the absence of light, azobenzene derivatives were isolated as the pure *E*-isomers.

**Scheme 6:**
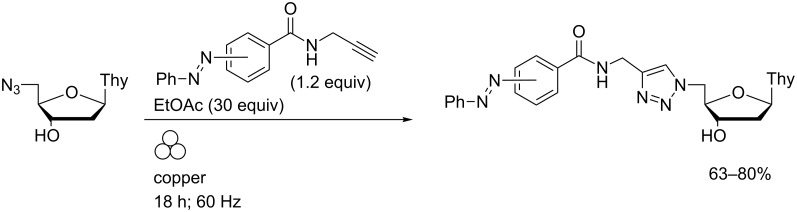
Azide–alkyne cycloaddition reactions performed in a copper vessel in a MBM.

In their original report, Sharpless and co-workers described the use of copper turnings to promote a regioselective azide–alkyne [3 + 2]-cycloaddition ("click") reaction over 24 hours [43]. High-speed ball milling using a custom-made copper vial and copper ball enabled efficient reaction between propyne-derivatised photoswitches and an azidodeoxynucleoside click partner (Scheme 6) [44].

**Figure 3:**
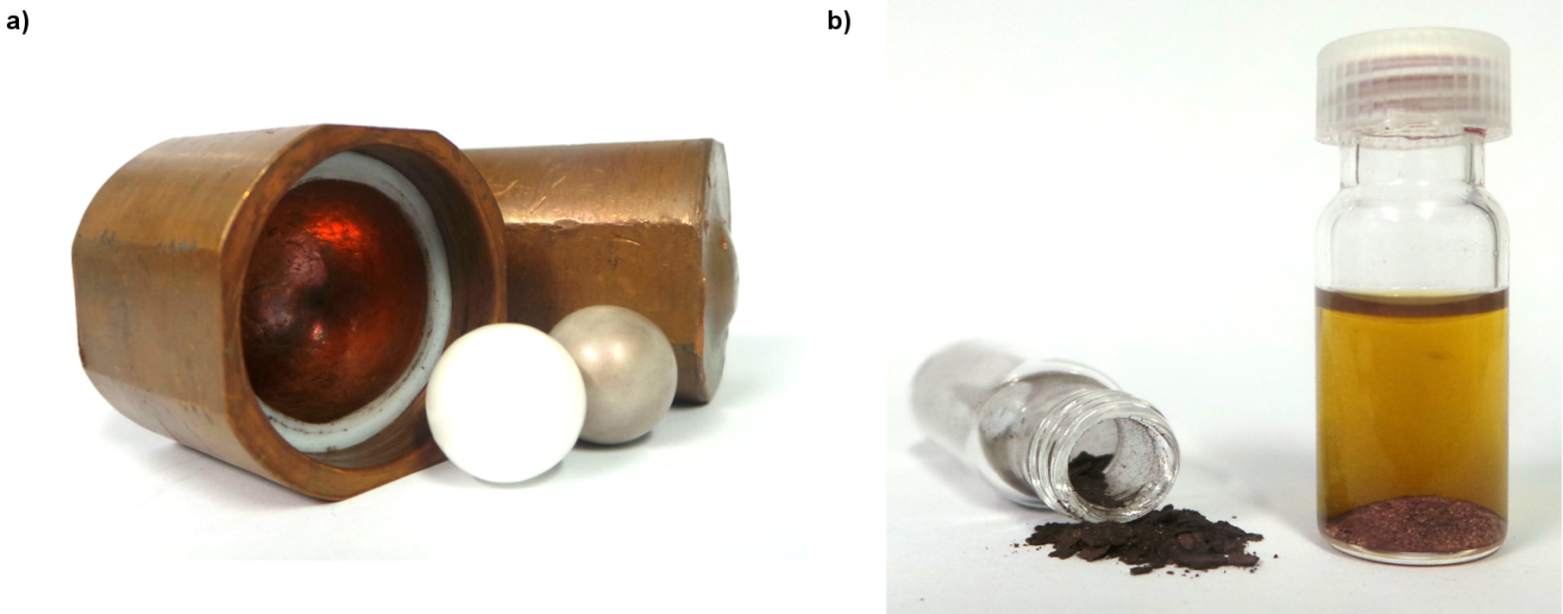
a) Custom-machined copper vessel and zirconia balls used to perform CuAAC reactions (showing: upper half of vessel with PTFE insert (front), pristine ZrO_2_ ball, used ZrO_2_ ball and lower half of vessel showing deformation of the metal). b) Crude solid ball mill click reaction mixture after removal from copper vessel (left) and during extraction of pure product with DMSO (right).

In contrast to the solution-phase (Cu(I)-promoted) reactions, no contamination of the ball milled products by copper salts was found. In an attempt to expedite the LAG reaction, millimol-scale reactions between the *p*-azobenzene-appended alkyne and 5′-azido-5′-deoxythymidine were attempted in a more capacious copper vessel with a 15 mm diameter zirconia ball (Figure 3). Clean and complete click reactions were achieved within 40 minutes at 25 Hz in the presence of ethyl acetate although the integrity of the vessel was compromised and significant levels of metallic copper were removed from the walls during work-up.

**Scheme 7:**
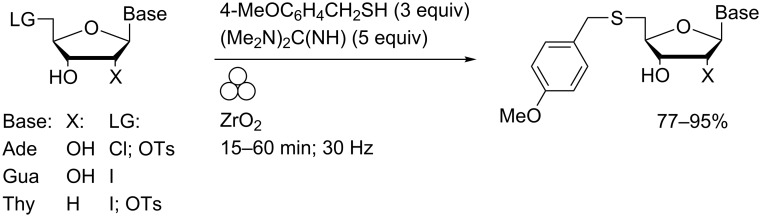
Thiolate displacement reactions of nucleoside derivatives in a MBM.

Expeditious displacement of tosylate or halides from 5′-derivatised nucleosides was achieved using chalogenate nucleophiles in a mixer ball mill using zirconia components [22]. Highly efficient transformations to the corresponding 4-methoxybenzyl thioethers were achieved in 15–60 minutes such that pure products could be isolated without the need for chromatography (Scheme 7). Of particular note was the absence of any observable intramolecular cyclisation of the unprotected purine nucleoside derivatives typical of solution-phase reactions using such substrates.

**Scheme 8:**
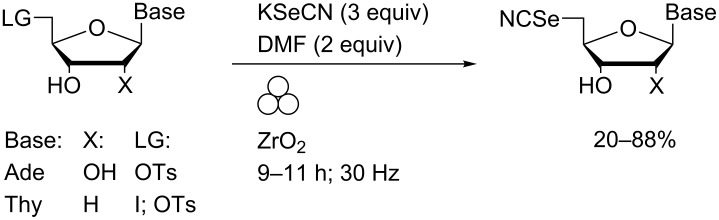
Selenocyanate displacement reactions of nucleoside derivatives in a MBM.

More variable yields were obtained using potassium selenocyanate which required grinding in the presence of DMF to promote the reaction with adenosine or thymidine derivatives (Scheme 8). No reaction of 5′-chloro-5′-deoxyadenosine was observed.

Under these conditions, cyclisation of 5′-tosyladenosine was inferred although rapid and clean reaction of 5′-iodo-5′-deoxyguanosine was apparent in the absence of added solvent – the product from this latter reaction rapidly decomposed during work-up in solution.

**Scheme 9:**
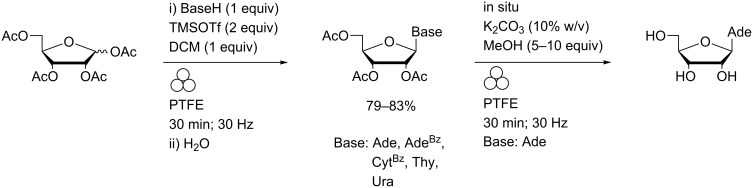
Nucleobase glycosidation reactions and subsequent deacetylation performed in a MBM.

Regioselective and stereoselective glycosidation of adenine, *N*^6^-benzoyladenine, *N*^4^-benzoylcytosine, thymine and uracil to the corresponding β-*N*^9^-purine or β-*N*^1^-pyrimidine ribosides was achieved on gram scales under Vorbrüggen-type conditions using LAG (Scheme 9) [45].

Yields were slightly enhanced following presilylation of the bases in solution prior to ball milling and under these conditions, the corresponding protected 6-chloropurine riboside could also be accessed. Multiple products were formed from *N*^2^-iso-butyrylguanine and hypoxanthine but cytosine remained untransformed. In situ deprotection of 2′,3′,5′-tri-*O*-acetyladenosine was also claimed. This chemistry has also been applied to the preparation of a library of ribosylated nicotinamide and nicotinic acid ester derivatives in a mortar grinder or using mixer or planetary ball mills [46–47]. Reaction scales up to 40 g were described and the conditions developed enabled exclusive formation of the β-anomer of nicotinamide riboside (NR) in the absence of toxic bromide salts.

#### Preparation and reactions of nucleotides and their analogues

**Scheme 10:**
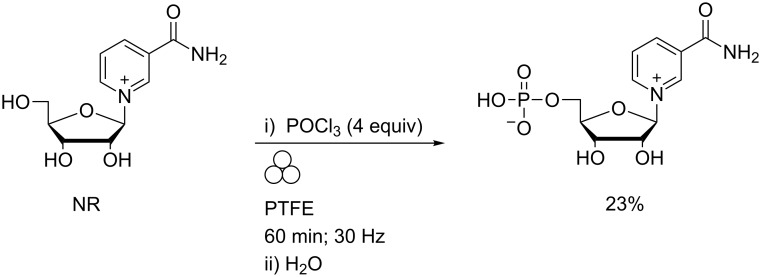
Regioselective phosphorylation of nicotinamide riboside in a MBM.

Phosphorylation of NR on gram-scales using POCl_3_ (e.g., Scheme 10), monoalkyl phosphorodichloridates or dialkyl phosphoromonochloridates in the absence of solvent has been reported [48].

**Scheme 11:**
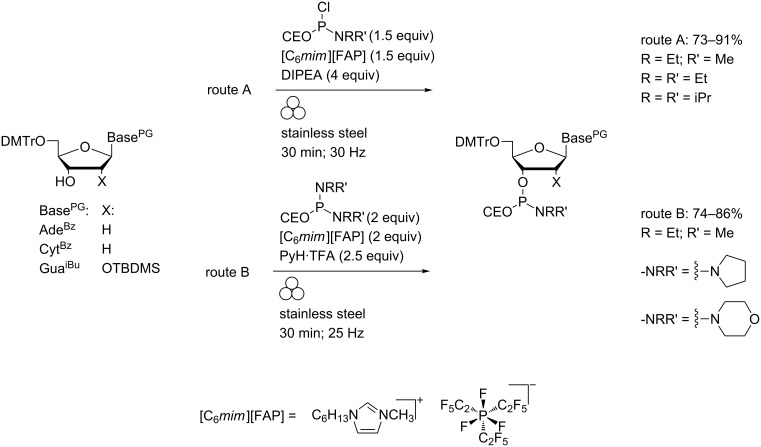
Preparation of nucleoside phosphoramidites in a MBM using ionic liquid-stabilised chlorophosphoramidites (route A) or phosphorodiamidites (route B).

Migaud and co-workers prepared highly water-sensitive phosphitylating agents directly from PCl_3_ in low viscosity ionic liquids derived from the tris(pentafluoroethyl)trifluorophosphate anion (e.g., [C_6_*mim*][FAP]) and subsequently used the crude chlorophosphoramidite or phosphorodiamidite products to effect nucleoside phosphitylations using LAG (Scheme 11) [49–50].

**Scheme 12:**
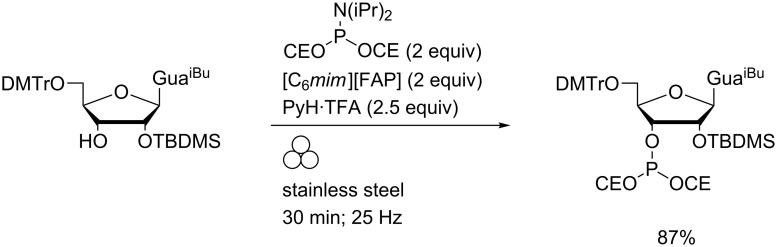
Preparation of a nucleoside phosphite triester using LAG in a MBM.

In the absence of grinding, addition of a molecular cosolvent was required due to the low solubility of substrates in the ionic liquids (<10 mM) which rendered the phosphitylating agents prone to hydrolysis. Highly reactive phosphoramidite derivatives of low molecular weight amines could be isolated by this route (on 40–60 mg scales). Under the same conditions, coupling of bis(2-cyanoethyl)diisopropylaminophosphoramidite with a partially-protected guanosine derivative to the corresponding phosphite triester was also effected (Scheme 12).

**Scheme 13:**
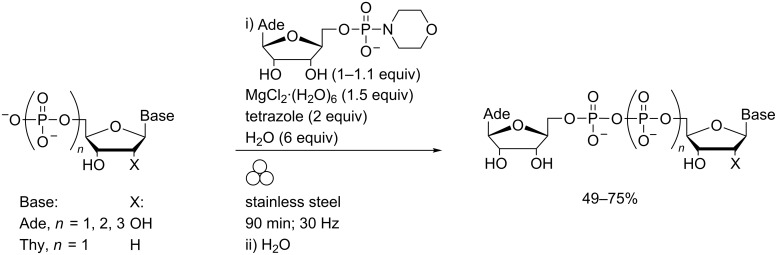
Internucleoside phosphate coupling linkages in a MBM.

Phosphate coupling using nucleoside phosphoromorpholidates is well established [51] but the reaction times are typically in the order of days. Recent developments in this field which yield pyrophosphate bonds more rapidly have been comprehensively reviewed by Peyrottes and co-workers [52] but in all cases, efficient coupling has been predicated on strictly controlling the water content of the reaction mixture. In contrast, LAG in the presence of water enabled the coupling of adenosine-5′-monophosphoromorpholidate with the sodium salts of 5′-phosphorylated nucleosides without any predrying and in the presence of acidic promoters and water gave complete reaction within 90 minutes (Scheme 13) [53].

**Scheme 14:**
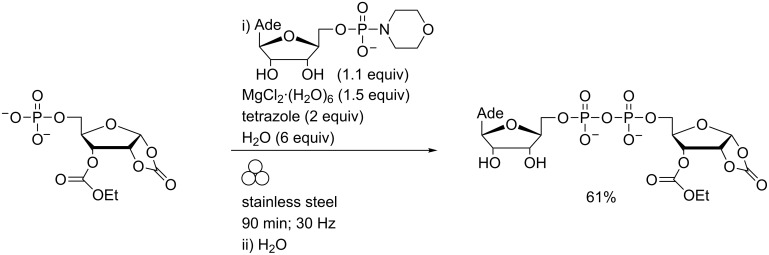
Preparation of ADPR analogues using in a MBM.

In this original report, the preparation of nicotinamide adenine dinucleotide (NAD) and adenosine diphosphate ribose (ADPR) was also described. Subsequently, this methodology was applied to the preparation of a library of six ADPR carbonate derivatives in 23–68% yields (e.g., Scheme 14) and tested as sirtuin inhibitors [54].

**Scheme 15:**
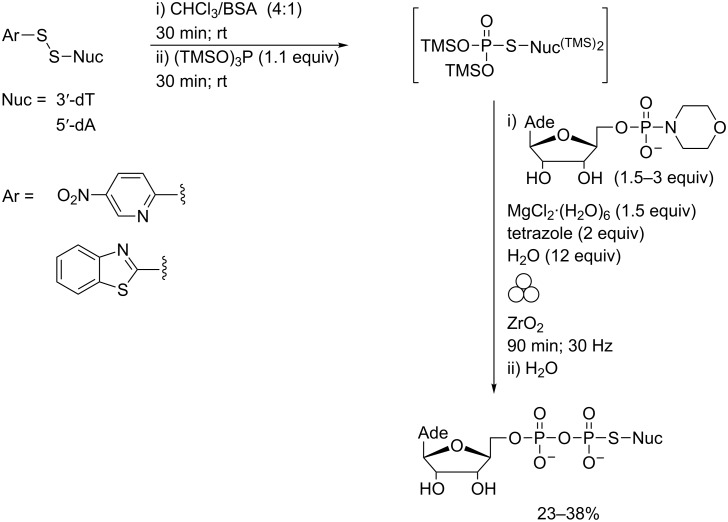
Synthesis of pyrophosphorothiolate-linked dinucleoside cap analogues in a MBM to effect hydrolytic desilylation and phosphate coupling.

The efficiency of phosphate coupling under mechanochemical conditions was exploited to prepare pyrophosphorothiolate-linked dinucleoside cap analogues. Such materials had previously been inaccessible via this route due to the lability of intermediate phosphorothiolate monoesters under acidic conditions [55]. In contrast, the corresponding persilylated derivatives were found to be relatively stable under anhydrous conditions and could be readily prepared via Michaelis–Arbusov (M–A) chemistry (Scheme 15) [23,56].

Transfer of crude M–A reaction mixtures to a zirconia ball mill vessel and removal of volatiles enabled the concomitant partial hydrolytic desilylation of the monoester and phosphate coupling to AMP-morpholidate to be effected in one pot using LAG. Both 3′,5′- and 5′,5′- internucleoside linkages were prepared using this route.

### Mechanochemical transformations of nucleosides and related materials involving non-covalent bonds

#### Dissociative processes for DNA and RNA isolation

The lack of free-volume within double-stranded DNA at low hydration levels leads to limited ice formation even under cooling in liquid air [57]. In contrast, cold denaturation of globular proteins at such temperatures is almost ubiquitous [58]. Furthermore, large conformational reorientation of protein domains can be initiated at 30 pN compared with DNA which requires ca. 150 pN of highly directional force to bring about duplex melting [26]. Early recognition of these differences (even without a full understanding of their molecular origins) by pioneers in the field contributed to the development of DNA purification which featured mechanochemistry at low temperatures [59].

Miescher reported grinding the solid residues from defatted salmon sperm heads with dilute HCl (0.5%) to effect such a separation and was able to report elemental analysis of nuclein with a phosphorus content (9.6%) close to that of the theoretical protein-free value [60–61].

Due to the accessibility of calf thymus and its high DNA content, much of the further work on large-scale extraction of "sodium nucleate" was performed using this tissue. Although details of the grinding actions employed were not always fully described, intensive mechanochemical processing (especially at low temperature) enabled the (frozen) fresh tissue to be powdered and in a subsequent step to bring about disaggregration of protein–DNA complexes at acidic pH. Early reports predominantly featured hand grinding in a mortar but could also include crushing with a glass rod and be supplemented by the use of a meat mincer and subsequently by an electrical blender [59,62–64]. The pure polymeric material isolated by these routes played a considerable role in revising Levene’s tetranucleotide colloid hypothesis as he conceded in 1938 [65]. This culminated in Schwander and Signer isolating eight grams of pure material [63] from which high quality X-ray diffraction images (including "Photograph 51") [66] were obtained and its double-helical structure evinced [67].

Parallel to Miescher’s work on salmon sperm, Kossel reported the isolation of ribonucleotide-derived material from yeast RNA in 1879 using mechanical disaggregation [68].

Altmann developed a more generalised method for isolating either RNA or DNA from a variety of tissues and organisms during which crude mixtures with protein were ground to a fine powder with 1:1 alcohol/6% HCl (aq) and subsequently triturated with pure alcohol and then ether [69]. Typically, RNA depolymerisation (especially when in contact with metal components) would be observed during these operations [70] although isolation of infectious viral RNA from frozen carcinoma tissue following grinding was reported in 1957. More recently, RNA was extracted from both Gram-negative and Gram-positive bacteria by hand grinding in a mortar with phenol under cooling with liquid nitrogen [71] .

**Figure 4:**
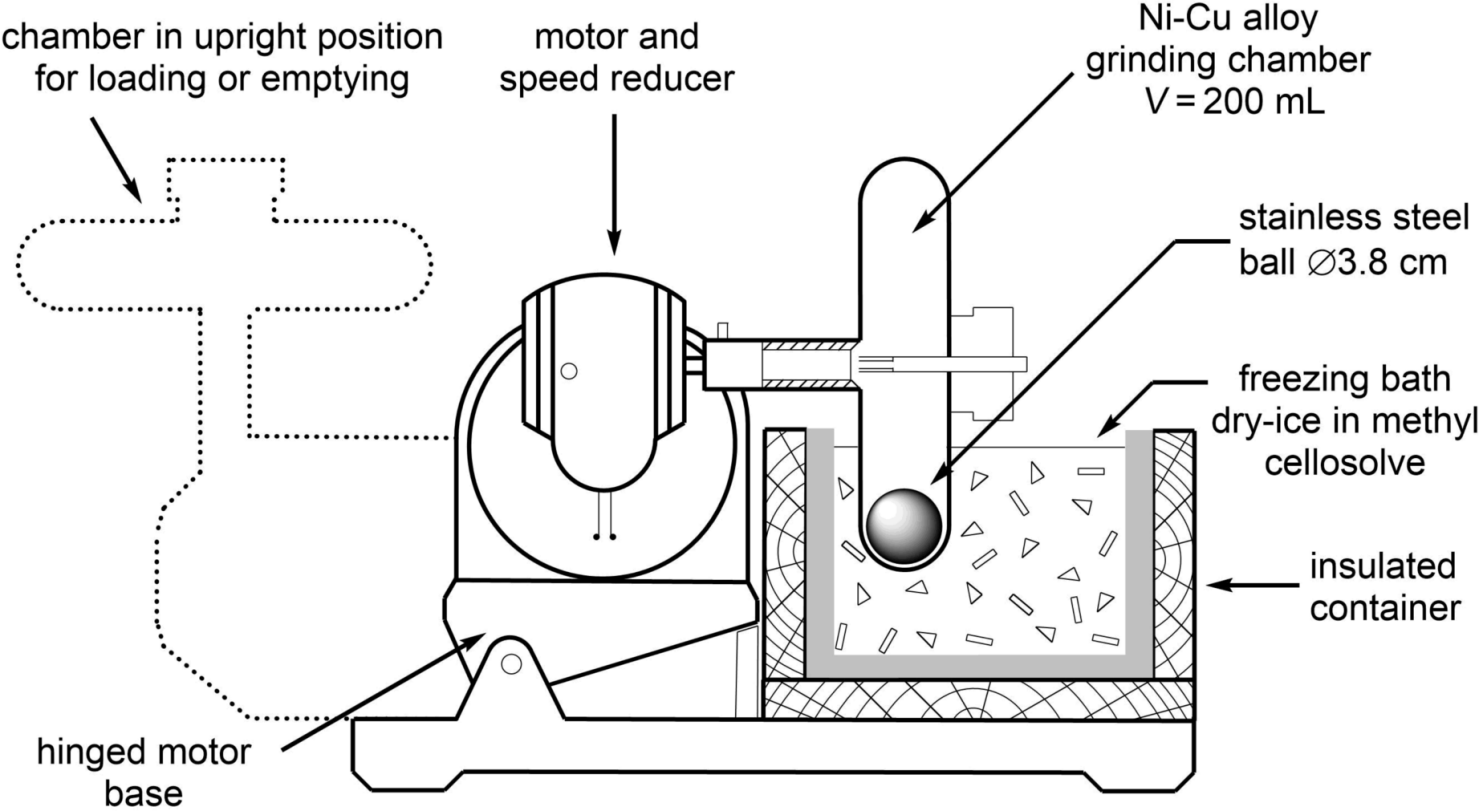
Early low temperature mechanised ball mill as described by Mudd et al. – adapted from reference [78].

The reproducibility of studies requiring arduous mechanochemical operations to be performed by hand lead to the rapid uptake of mechanisation for performing grinding actions by workers in this field. Behrens‘ procedure for isolating nuclei from calf heart included three separate mechanochemical operations first using a meat grinder, then a mortar and pestle and finally a mechanised ball mill in which a one litre flask containing 800 g of "glass pearls" was shaken at 3 Hz prior to subsequent trituration with benzene and carbon tetrachloride [72]. A subsequent development of this procedure included liquid-assisted grinding in a one litre porcelain jar which was rotated at 110 rpm for 24–48 hours with up to 1.4 kg of grinding stones (15–20 mm in diameter), 100 g of dried tissue powder and petroleum ether (200–450 mL) [73–75]. As early as 1903, low temperature grinding was applied to disrupting refractory mycobacteria (using zirconia components under cooling in liquid air) [76] and subsequently mechanised using a steel ball mill (at −78 °C) [77] (e.g., Figure 4) [78].

Subsequently, this technology has been developed to allow larger scale ball milling of tissue samples (including at liquid nitrogen temperatures) and smaller scale "bead beating" in disposable plasticware. Depending upon the nature of the biological material and target sequence, grinding balls made of zirconia, garnet, glass or steel enable isolation and quantification of DNA or RNA from different sources [79]. Recently, a micro total analysis system was fabricated incorporating a 15 μL cell lysis chamber containing glass beads (30–50 μm) which were agitated using a membrane valve [80]. Within three minutes, almost complete disruption of Gram-positive bacteria was effected enabling downstream analysis by quantitative PCR.

#### Associative processes

Variable drug bioavailability associated with crystal and co-crystal polymorphism can be exacerbated if the solubility profiles of the API and coformer prevent solution-phase mixing. Under such circumstances, mechanochemistry can play a valuable role in improving both uniformity of dispersion and the screening rate of such polymorphs [10].

**Scheme 16:**
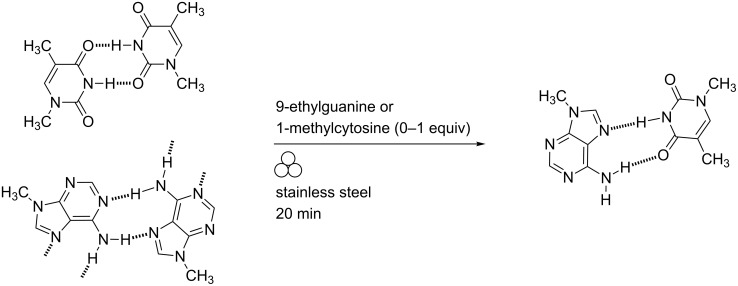
Co-crystal grinding of alkylated nucleobases in an amalgam mill (N.B. no frequency was recorded in the experimental description).

Etter and co-workers showed that solid-state grinding of equimolar quantities of 9-methyladenine and 1-methylthymine in an amalgam mill gave powder diffraction patterns consistent with the formation of Hoogsteen-type base-pairing (Scheme 16) [81].

No co-crystal formation was observed using 1-methylcytosine with 9-ethylguanine or other combinations which did not contain both adenine and thymine derivatives. The specificity of the Ade–Thy hydrogen bonding was not disrupted in the presence of non-interacting bases.

**Figure 5:**
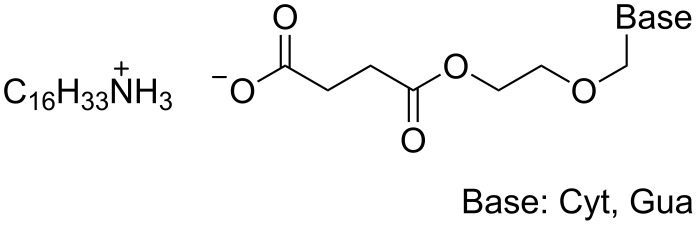
Materials used to prepare a smectic phase.

Tsiourvas and co-workers obtained a similar result after grinding an equimolar mixture of the hexadecylammonium salts of a succinylated acyclovir derivative (Figure 5) and its cytosine congener in an undefined "vibrator mill" at room temperature. However, above 80 °C a solid-state transition was observed in which base-pairing was inferred and upon further heating gave a smectic phase [82].

**Figure 6:**
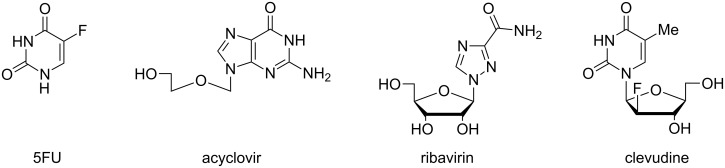
Structures of 5-fluorouracil (5FU) and nucleoside analogue prodrugs subject to mechanochemical co-crystal or polymorph transformation.

Using a mixer ball mill, Vogt and co-workers ground 5-fluorouracil (5FU, Figure 6) and thymine over a wide stoichiometry range either under dry conditions or in the presence of a variety of organic solvents [83]. Liquid-assisted grinding of mixtures containing 50–90 mol % 5FU at 30 Hz for 30 min gave homogenised solid solutions using two drops of acetonitrile.

LAG of a 1:1 mixture of 5FU/4-hydroxybenzoic acid using a variety of liquids yielded co-crystals exhibiting polymorphism which was dependent upon the polarity of the added liquid [84]. Co-crystals of structurally-related carboxylic acids with 5FU prepared using LAG in a MBM in the presence of water exhibited enhanced membrane permeability compared with the pure API [85]. The preparation of co-crystals of 5FU with other API’s (imatinib [86] and piperazine [87]) using LAG has also been reported.

Solid dispersions of acyclovir (20%) in neutral carriers (chitosan, hydroxypropylmethyl cellulose K100M^®^ or Pluronic F68^®^) were prepared in a mixer ball mill over three hours [88]. All dispersions displayed antiviral activity and enhanced aqueous dissolution rates. The Pluoronic F68^®^ dispersion displayed enhanced transport rates across a model intestinal cell monolayer.

The conversion of a stable ribavirin polymorph R-II into its metastable enantiotrope R-I has been investigated using mechanochemistry [89–90]. LAG in an improvised planetary mill using lead balls gave limited phase conversion [89] but dry milling R-II in a commercial mixer mill at 30 Hz gave 100% conversion within 15 minutes [90]. Three crystal polymorphs of the antiviral nucleoside prodrug clevudine were characterised and a large scale preparation of the most stable form from commercial material was performed using LAG in a mortar [91].

**Scheme 17:**
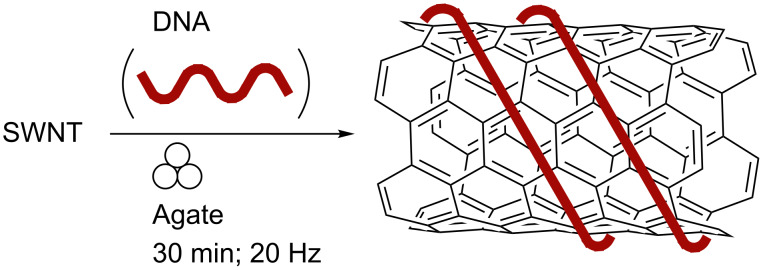
Preparation of DNA-SWNT complex in a MBM.

Geckeler and co-workers described the efficient preparation of both multi-walled and single-walled carbon nanotubes (CNTs) by grinding these materials in a mixer ball mill using agate components (Scheme 17) [92].

In the absence of CNTs, DNA cleavage to a uniform size was found. Ball milling in the presence of monoribonucleotides has also been investigated as a method for solubilising single-walled carbon nanotubes [93]. In the presence of guanosine-5′-monophosphate, 78% of the SWNT (0.78 mg mL^−1^) was dissolved but attempted removal of iron contamination from this material by treatment with acid gave a "viscous precipitate".

Formation of cyclodextrin–drug inclusion complexes can be accelerated using mechanochemistry [94] and Rajamohan and co-workers described using a mortar and pestle to effect LAG of β-cyclodextrin with either inosine [95] or cytidine [96] in the presence of water. Weak complex formation was inferred by powder XRD for cytidine.

## Conclusion

Access to reliable and reproducible mechanised grinding has generated an upsurge in interest in mechanochemistry for a variety of chemical applications over the past decade. As a frontier science, theoretical models of reactivity under the action of mechanical forces are rapidly undergoing revision in the light of results available both from observations at a molecular scale [97] and from in situ monitoring of bulk-scale reactions [98]. The limited work relating to the chemical transformation of nucleoside and nucleotide substrates has been mainly focussed upon exploiting the (lack of) solvent requirements. This can allow access to unprecedented mechanochemical reaction pathways which would otherwise be unavailable through conventional solution-phase chemistry. This has included the preparation of pharmaceutical grade NR exclusively as the β-anomer and in the absence of bromide contamination [99] and in situ hydrolytic unmasking of labile phosphorothiolate monoesters prior to rapid phosphate coupling. However, at the interface between biology and chemistry, the use of grinding to effect force-induced (mainly) dissociative reactions has a considerably longer heritage and it can be argued that Buchner’s Nobel Prize in Chemistry was the first in this field. Although speaking from a more theoretical stand-point following observations on the infectivity of bacteriophages, Muller discussed the concept of genetic manipulation using (mechano)chemistry in 1922, commenting "perhaps we may be able to grind genes in a mortar and cook them in a beaker" [100].

Dubinskaya reviewed early investigations into the grinding and stretching of polypeptides and proteins which showed rapid loss of enzyme activity at 80 K [101]. In contrast, more recent reports, in which both native and immobilised enzymes were ground at higher temperatures, demonstrated efficient mechanoenzymatic transformations of amino acid [102–107], cellulose [108] or model lignin [109] substrates. Implicit within these studies is the resilience of chiral centres within both substrate and catalyst towards epimerisation during ball milling. This was also explicitly demonstrated by the Nagy lab in the context of developing models for the origin of non-racemic amino acid content within meteorites [110–111]. A role for mechanochemistry in understanding the origins of biochirogenesis is suggested by phase separation of co-crystals of the D- and L-enantiomers of malic acid in the presence of L-tartaric after grinding the racemate [112]. Heinicke briefly summarised early investigations into potential prebiotic α-amino acid preparation under “tribochemical stress” in the presence of transition metals [113] and more recently, Hernández and co-workers demonstrated that an efficient Strecker-type reaction could be effected in a ball mill using catalytic anhydrous ferricyanide in the presence of silica [114]. From a theoretical perspective, Hansma proposed that such mechanical energy could be supplied within moving mica sheets under high molecular crowding conditions [115].

In contrast, consideration of primordial nucleoside and nucleotide mechanochemistry has been much more limited with greater focus upon precursor mechanosynthesis under high-energy plasma conditions [116–117] or extra-terrestrial delivery of concentrated transition metals [118]. In this general context, although Orgel and co-workers describe transformation of adenine hydrochloride and D-ribose into the corresponding nucleoside α- (4%) or β- (3%) anomers following “thorough grinding” and heating of the mixture they do not distinguish between mechanochemistry and thermochemistry effects [119]. Considering recent investigations into low temperature ice eutectic phases as the incubators of early life [120] and separately, the effects of high hydrostatic pressures upon ribozyme activities [121–122], it is surprising how little consideration has been given to the role of nucleic acid mechanochemistry under prebiotic conditions. The capacity for stereoselective glycosidation, rapid phosphate coupling in the presence of water and also formation of specific base-pairing interactions have all been demonstrated in a ball mill and may facilitate understanding of the early appearance of life in the Hadean/Archean Eon.

## Abbreviations

**Table 1:** List of abbreviations.

5FU	5-fluorouracil
Ade	*N*^9^*-*adeninyl
Ade^Bz^	*N*^6^-benzoyl-*N*^9^-adeninyl
ADPR	adenosine diphosphate ribose
AMP- morpholidate	adenosine 5′-monophosphoromorpholidate
Base	nucleobase (Ade, Cyt, Gua, Hyp, Thy or Ura)
Boc	*tert*-butyloxycarbonyl
BSA	*N,O*-bis(trimethylsilyl)acetamide
[C_6_*mim*]	1-hexyl-3-methylimidazolium
CE	2-cyanoethyl
CNT	carbon nanotube
CuAAC	copper-assisted azide alkyne cycloaddition
Cyt	*N*^1^-cytosinyl
Cyt^Bz^	*N*^4^-benzoyl-*N*^1^*-*cytosinyl
dA	deoxyadenosinyl
DABCO	1,4-diazabicyclo[2.2.2]octane
DIPEA	*N*,*N*-diisopropylethylamine
DMAP	4-*N,N*-(dimethylamino)pyridine
DMTr	4,4′-dimethoxytrityl
dT	deoxythymidinyl
[FAP]	tris(pentafluoroethyl)trifluorophosphate
Gua	*N*^9^-guaninyl
Gua^iBu^	*N*^2^-isobutyryl-*N*^9^-guaninyl
HMDS	hexamethyldisilazane
Hyp	*N*^9^-hypoxanthinyl
LAG	liquid-assisted grinding
M–A	Michaelis–Arbuzov
MBM	mixer ball mill
MMTr	4-methoxytrityl
NAD	nicotinamide adenine dinucleotide
NR	nicotinamide riboside
PCR	polymerase chain reaction
PG	protecting group
PTFE	polytetrafluoroethylene
Py	pyridine
SWNT	single-walled carbon nanotube
TBAB	tetra-*n*-butylammonium bromide
TBDMS	*tert*-butyldimethylsilyl
TFA	trifluoroacetate
Thy	*N*^1^-thyminyl
Tr	trityl
Ura	*N*^1^-uracilyl
